# Development and validation of GLVS (Generic Laparoscopic Video Scoring System), a tool for assessment of laparoscopic skills in gynaecology using videotaped procedures: Are edited videos representative of their full-length version?

**DOI:** 10.52054/FVVO.15.2.085

**Published:** 2023-09-24

**Authors:** S Khazali, A Bachi, T.T. Carpenter, A Moors, K Ballard

**Affiliations:** Centre for Endometriosis and Minimally Invasive Gynaecology (CEMIG London), HCA The Lister Hospital, Chelsea Bridge Road, London, United Kingdom, SW1W 8RH; Royal Holloway, University of London, Egham, United Kingdom, TW20 0EX; Ashford and St. Peter’s Hospital NHS Foundation Trust, Department of Obstetrics and Gynaecology, Guildford Road, Chertsey, United Kingdom, KT16 0PZ; Poole Hospital NHS Trust, Department of Obstetrics and Gynaecology, Longfleet Road, Poole, Dorset, United Kingdom, BH15 2JB; Princess Anne Hospital, Department of Obstetrics and Gynaecology, Coxford Road, Southampton, United Kingdom, SO16 5YA; University of Surrey, Postgraduate Medical School, Daphne Jackson Road, Manor Park, Guildford, Surrey, GU2 7WG

**Keywords:** Assessment tool, laparoscopic skills

## Abstract

**Background:**

Anonymized videotaped endoscopic procedures can be used for the assessment of surgical competence, but a reliable non-procedure-specific scoring system is needed for gynaecology.

**Objectives:**

To design and evaluate the validity of the Generic Laparoscopic Video Scoring System (GLVS), a novel tool in the assessment of various gynaecological laparoscopic procedures.

**Material and Methods:**

Seventeen anonymized unedited video recordings of various gynaecological laparoscopic procedures and the 4-minute-long edited versions of the same videos were independently scored by two experts, twice, using GLVS.

**Main outcome measures:**

Internal consistency reliability, test-retest, and inter-rater reliability of GLVS. We also compared the scored achieved by edited videos with those of the full-length version of the same videos.

**Results:**

The mean score achieved by 4-minute-long edited videos was similar to that of the unedited version (p= 0.13 - 0.19). There was excellent correlation between the pooled scores for edited and unedited versions (intra- class correlation coefficient = 0.86). GLVS had excellent internal consistency reliability (Cronbach’s alpha 0.92- 0.97). Test-retest and inter-rater reliability were generally better for edited 4-minute-long videos compared to their full-length version. Test-retest reliability for edited videos was excellent for scorer 1 and good for scorer 2 with intra-class correlation coefficient (ICC) of 0.88 and 0.62 respectively. Inter-rater reliability was good for edited videos (ICC=0.64) but poor for full-length versions (ICC= -0.24).

**Conclusion:**

GLVS allows for objective surgical skills assessment using anonymized shortened self-edited videos of basic gynaecological laparoscopic procedures. Shortened video clips of procedures seem to be representative of their full-length version for the assessment of surgical skills.

**What's new?:**

We devised and undertook a validation study for a novel tool to assess surgical skills using surgical video clips. We believe this addition clearly delineates the unique contributions of our study.

## Introduction

The objective assessment of surgical skills is complex and multiple assessment tools have been developed. However, like most clinical assessment tools, they have a potential to introduce biases such as leniency bias and halo effect. Fundamentals in Laparoscopic surgery (FLS) has been widely used to test laparoscopic surgical skills and is a prerequisite for board eligibility in general surgery and obstetrics and gynaecology (American Board of Obstetrics and Gynecology). A recent systematic review examining the validity evidence of FLS examination has shown to be mixed or lacking (Lerner et al., 2021). Another example is the Objective Structured Clinical Examination (OSCE) and Objective Structured Assessment of Technical Skills (OSATs) which have been in use for some time now (Faulkner et al., 1996; Martin et al., 1997; Regehr et al., 1998).

Video recordings offer a valuable opportunity for the assessment of laparoscopic surgical skills. The outcome of its assessment differs to that of a live setting (van Hove et al., 2010) as videos can be anonymized and independently scored by more than one person or securely uploaded on the Internet and kept for records after scoring. Analysis of video-recorded procedures using generic global rating scales have been shown to have good correlation between a surgeon’s skills and patient outcome (Martin et al., 1997; Vassiliou et al., 2005; Vaidya et al., 2020).

However, a valid, generic, relevant, and easy- to-use scoring system is needed for gynaecology. Various systems have been proposed for scoring laparoscopic videos. Most of these have either been tested for assessing a specific and short part of certain procedures or for bench models in the laboratory (Scott et al., 2000b; Dath et al., 2004; Vassiliou et al., 2005; Swift and Carter, 2006; Jabbour and Sidman, 2011; Husslein et al., 2015; Vedula et al., 2017; Zia et al., 2018; Funke et al., 2019).

The objective of this study was to develop and investigate the usability and reliability of a new assessment tool, Generic Laparoscopic Video Scoring (GLVS) system for assessment of surgical skills, using recordings of basic laparoscopic gynaecological procedures and to compare the scores between the full-length (unedited) and the shortened (edited) versions of the same procedure.

GLVS is a novel tool developed for the purpose of surgical video assessment. It was created by the author (S.K.) as part of his master’s degree dissertation in 2009, but this is the first study designed to assess its validity and effectiveness in this particular role. The GLVS tool has not been used or evaluated prior to this study. The aim of the study was to test and present this novel tool to the scientific community, providing a comprehensive assessment of its reliability in scoring surgical videos.

## Methods

This study was conducted at the University of Surrey, UK, following ethical approval from the ethical committee of Faculty of Health and Medical Sciences, University of Surrey (Ref. EC/2010/01). Students taking part in a master’s degree program in advanced Gynaecological Endoscopy were required to submit three 4-minute-long videos of specific standard gynaecological procedures as part of an OSCE exam. For this study, the students were asked to provide the full-length recordings of the same procedures in addition to the 4-minute edited recordings. The procedures were laparoscopic salpingectomy for ectopic pregnancy, laparoscopic salpingo-oophorectomy, laparoscopic ovarian cystectomy, and laparoscopic adhesiolysis. Each surgical procedure was performed in its entirety by the same surgeon.

The students were either in the final two years of their specialty training, undertaking a fellowship, or were recently qualified gynaecologist. All surgeons provided written consent to use their videos for this study.

**Figure 1:**
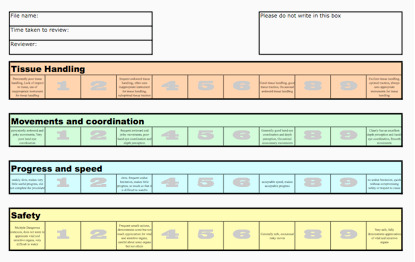
Generic Laparoscopic Video Scoring system (GLVS).

All videos were scored independently by 2 reviewers using the GLVS system. These reviewers were both gynaecological laparoscopic experts working as consultants at tertiary referral centres, with vast experience in performing and teaching gynaecological endoscopic surgery. The reviewers were provided with verbal instructions on how to use GVLS to score the recorded procedures but were not provided with any hands-on training in the use of GLVS. A paper-based scoring sheet was pre- printed for each video (Figure 1). In the first round of scoring, the reviewers measured the time taken to review the unedited version of each video. When reviewing the full-length version, the assessors were allowed to fast-forward through the video if they wished; for example, when the surgeon is waiting for an instrument. The 4-minute edited videos had to be viewed in full and no fast-forwarding was permitted.

All recordings were anonymised. The reviewers were therefore blinded to the training level and identity of the primary surgeon, avoiding introduction of bias.

### The GLVS scoring system

Drawing on Global Operative Assessment of Laparoscopic Skills (GOALS) devised by Vassiliou et al. (2005) we designed the Generic Laparoscopic Video Scoring system (GLVS). The scoring system contains a standard assessment of four components:

1. tissue handling (TH), 2. movements and coordination (MC), 3. progress and speed (PS) and 4. safe practice (SA). Each component of GLVS is assessed using an 11-point visual analogue scale ranging from 0-10. There are four descriptive anchor points at scores of 0, 3, 7 and 10. The anchor points were as below:

#### Tissue Handling (TH)

0- Persistently poor tissue handling, lack of respect to tissue, use of inappropriate instrument for tissue handling

3- Frequent awkward tissue handling, often uses inappropriate instrument for tissue handling, suboptimal tissue traction

7- Good tissue handling, good tissue traction, occasional awkward tissue handling

10- Excellent tissue handling, optimal traction, always uses appropriate instruments for tissue handling

#### Movements and Coordination (MC)

0- Persistently awkward and jerky movements, very poor hand-eye coordination

3- Frequent awkward and jerky movements, poor hand-eye coordination, and depth perception

7- Generally good hand eye coordination and depth perception, occasional unnecessary movements

10- Clearly has an excellent depth perception and hand-eye coordination, smooth movements

#### Progress and speed (PS)

0- Unduly slow, makes very little useful progress, did not complete the procedure

3- Slow, frequent undue hesitation, makes little progress, so much so that it is difficult to watch

7- Acceptable speed, makes acceptable progress

10- No undue hesitation, quick without compromising safety or respect to tissue

#### Safe practice (SA)

0- Multiple Dangerous instances, does not seem to appreciate vital and sensitive organs, very difficult to watch

3- Frequent unsafe actions, demonstrates some but not much appreciation for vital and sensitive organs, careful about some organs but not others

7- Generally safe, occasional risky moves

10- Very safe, fully demonstrates appreciation of vital and sensitive organ

With our emphasis on the need to determine whether the operator is demonstrating safe practice, the ‘safe practice’ score is given double weight. For ease of use, we wanted to have a final score from 0 -100, rather than 0-50 and so the final GLVS score is multiplied by two. Therefore, GLVS score is calculated as below:

GLVS score = (TH+MC+PS+SA+SA) x 2

Where TH = Tissue handling, MC= Movements and coordination, PS = Progress and speed, SA = Safe practice.

The main outcome measures of this study were inter-rater and intra-rater reliability. To evaluate for inter-rater reliability, the laparoscopic experts independently scored all the videos (edited and unedited). The reviewers were not in touch during the scoring period and did not communicate in any way either before or during the scoring process.

To evaluate for intra-rater reliability and test- retest reliability, each scorer scored all the videos on two occasions. To reduce the chances of score recall between the two rounds of scoring, the videos were scored at least three weeks apart and the file names were changed for the second round of scoring.

To test whether the 4-minute edited videos were scored similarly to the full-length videos, the scorers independently scored both unedited and edited videos, each identified by a different randomly generated file name.

Cronbach’s alpha was used to evaluate internal consistency of the GLVS. The inter-rater reliability was evaluated each time the two laparoscopic experts used the GLVS. Intra-class correlation coefficient (ICC) and Cronbach’s alpha were used to assess both the inter- and intra-rater reliability. ICC <0.4 was considered to reflect poor correlation, 0.4-0.59 fair correlation, 0.6-0.79 good correlation and >0.8 reflected an excellent correlation.

To compare the means of scores for edited and unedited videos, data was first tested using the Shapiro-Wilk test to see if they were normally distributed. The student t-test was used in normally distributed data whereas the Mann-Whitney U test was used for data which did not have a normal distribution.

To test whether each scorer in each round scored the edited version of each unedited video similarly, Cronbach’s alpha and intra-class correlation coefficient (ICC) were calculated.

## Results

A total of 50 videos were received (25 edited and 25 unedited versions of the same recording). Of the 25 unedited videos, 5 files were corrupt and would not play, 1 was an incomplete video finishing halfway through the procedure and 3 were either not anonymized or difficult to anonymize as they had audio and there were multiple scenes showing the surgeon, the assistant, or the theatre staff. These videos and their edited versions were excluded.

A total of 34 videos (17 edited and 17 unedited version of the same recording) were scored by two experts. There were 4 ovarian cystectomies, 6 salpingectomies for ectopic pregnancy and 6 salpingo-oophorectomies (one, as part of a laparoscopically assisted vaginal hysterectomy) and 1 adhesiolysis. All recordings were complete, starting from the introduction of the laparoscope to the end of the procedure,

All the edited videos were exactly or just under 4 minutes long. The median time for the unedited videos was 31 minutes and 29 seconds, ranging from 13 minutes 22 seconds to 1 hour, 44 minutes and 3 seconds.

### Scores

GLVS score was calculated by adding the weighted scores of individual items (Total score = (TH+MC+PS+SA+SA) x 2)). The laparoscopic experts’ assessment scores ranged from 18/100 to 100/100 for the edited videos and from 12/100 to 100/100 for the full videos. Table I shows the details of GLVS scores for each round of scoring for each reviewer.

**Table I:** GLVS scores for each round of scoring for each reviewer.

			Min	Max	Mean	StDev
Scorer 1	Unedited	First round	24	100	62.8	21.03
		Second round	26	94	68.3	20.37
	Edited	First round	38	98	72.7	19.99
		Second round	34	100	73.2	21.04
Scorer 2	Unedited	First round	12	92	55.2	23.93
		Second round	18	80	55.6	16.94
	Edited	First round	20	94	65.1	19.40
		Second round	18	78	63.4	17.38

### Internal consistency reliability

The internal consistency of GLVS was found to be very high, with Cronbach’s alpha scores of between 0.923 and 0.974. This was calculated for every instance the scoring system was administered (edited and unedited videos, first and second round of scoring and for both scorers). Table II shows the internal consistency for the 8 times GLVS was administered. We also evaluated whether omitting any of the items within the scoring system would improve the reliability of the test. All items appeared to be worthy of retention. The greatest increase in Cronbach’s Alpha value came from omission of “Safety” in the second round of scoring when the first scorer scored the Unedited videos. This increase in reliability was only 0.03 increase in the Alpha value (0.934 to 0.964) and is therefore negligible.

**Table II:** Internal reliability in the eight instances GLVS was administered.

			Cronbach’s Alpha	Cronbach’s alpha if item deleted	Difference
Scorer 1	Unedited	First round	0.945	0.968*	0.023
		Second round	0.934	0.964*	0.030
	Edited	First round	0.974	0.975*	0.001
		Second round	0.923	0.947*	0.024
Scorer 2	Unedited	First round	0.972	0.984*	0.012
		Second round	0.958	0.968**	0.010
	Edited	First round	0.943	0.965*	0.022
		Second round	0.966	N/A***	N/A

*Omission of “Safety” increased the reliability; **Omission of “Tissue handling” increased reliability; ***Omission of none of the items increased reliability.

### Test-retest (intra-rater) reliability

GLVS had excellent intra-rater reliability when scorer 1 used it for edited videos (ICC=0.88). The intra-rater reliability was good for scorer 2 with both edited and unedited videos (ICC = 0.67 and 0.62 respectively). This reliability was fair for scorer 1, when scoring unedited videos (ICC=0.40). Table III shows the intra-rater reliability between the two rounds of scoring for different versions of videos separately.

**Table III:** Test-retest (intra-rater) reliability.

		Alpha	ICC
Scorer 1	Unedited (Round 1 vs Round 2)	0.578	0.406
	Edited (und 1 vs Round 2)	0.936	0.880
Scorer 2	Unedited (Round 1 vs Round 2)	0.806	0.675
	Edited (Round 1 vs Round 2)	0.771	0.627

### Inter-rater reliability

The GLVS had good inter-rater reliability when used for the edited videos (ICC=0.64) and poor inter-rater reliability when used for assessing the full-length videos (ICC=-0.24). When the mean of two rounds of scoring for each scorer was compared to the mean of two round for the other scorer, edited videos showed good inter-rater reliability (ICC = 0.60). Unedited videos, however, still showed poor inter-rater variability (ICC= 0.17)

When scoring unedited videos, scorers had poor inter-rater reliability in 3 of four possible combinations (ICC -0.24-0.17). Only when comparing the second round of both scorers, the inter-rater reliability was good (ICC=0.60).

Edited videos had fair to good inter-rater variability in three of the four combinations (ICC 0.54 – 0.64). Round 2 of both scorers showed poor inter-rater reliability (ICC = 0.38). Table IV summarizes the inter-rater reliability of GLVS in all possible combinations.

**Table IV:** Inter-rater reliability of GLVS in all possible combinations.

		Cronbach's Alpha	ICC
Unedited videos	Round 1 of both scorers	-0.630	-0.240
	Round 1 scorer 1 vs Round 2 scorer 2	0.261	0.150
	Round 2 of both scorers	0.755	0.606
	Round 2 Scorer 1 vs Round 1 Scorer 2	0.300	0.177
	Mean of two rounds for each scorer	0.292	0.171
Edited videos	Round 1 of both scorers	0.786	0.648
	Round 1 scorer 1 vs Round 2 scorer 2	0.704	0.543
	Round 2 of both scorers	0.553	0.382
	Round 2 Scorer 1 vs Round 1 Scorer 2	0.701	0.540
	Mean of two rounds for each scorer	0.750	0.600

### Comparison of scores between edited and unedited versions

**Figure 2:**
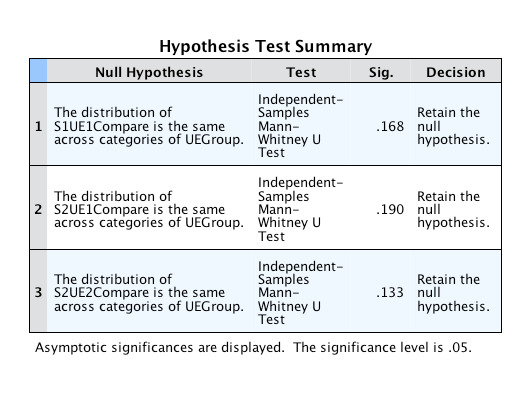
Mann-Whitney U test results for the pairs where one or both sides were not normally distributed.

**Figure 3:**
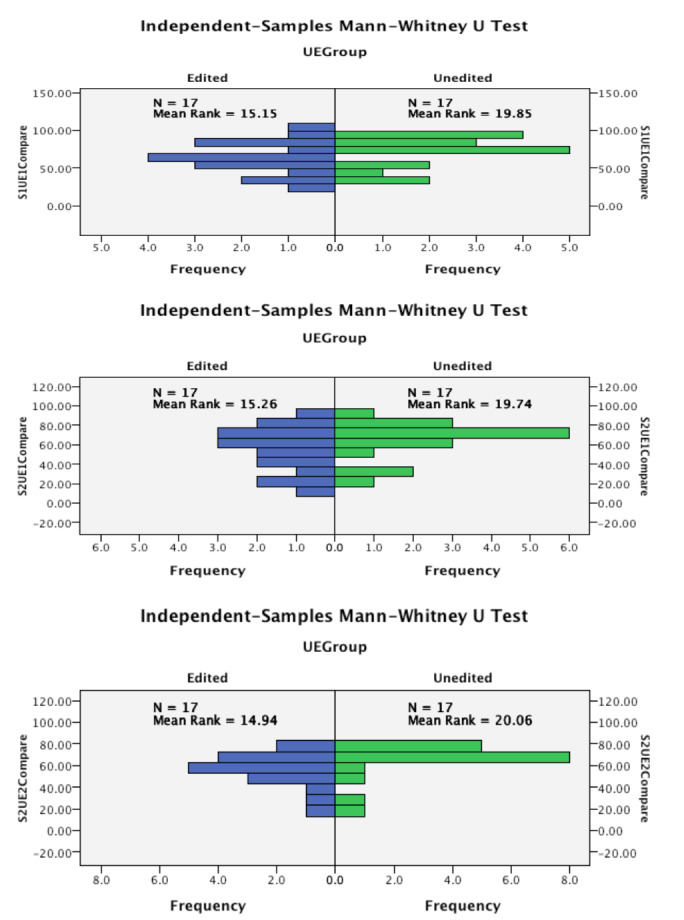
Mann-Whitney U test graphs showing no significant difference between edited and unedited scores.

To compare the scores obtained by edited and unedited version of the same video, we first tested to see if the total scores were normally distributed to choose the appropriate statistical test. Only in second round of scoring for scorer 1, both edited and unedited scores were normally distributed. We used student t-test to compare these two. In all other pairs either one or both sides of the comparison did not have a normal distribution and therefore Mann- Whitney U test was used to compare these pairs. There was no significant difference between edited and unedited videos in any of the rounds for any of the scorers (p=0.145 for student t-test and p=0.1333- 0.190 ranging between 0.133-0.190 where Mann- Whitney U test was performed). This means that the scorer did not tend to give a higher or lower score to edited videos compared to the unedited versions of the same video. Table V shows the differences in means of the edited and unedited videos and the result of student t-test (p=0.145) for round 2 of scorer 1 where both sides of the pair were normally distributed. The details of Mann-Whitney U test for other rounds are shown in Figure 2 and Figure 3. None of these showed significant difference between the scores for edited and unedited videos. Both scorers scored the two versions of each video similarly in the second round (ICC= 0.79 and 0.78 for scorer 1 and 2 respectively) showing almost excellent correlation but the correlation between the scores of the two versions was poor for the first round for both scorers (ICC= 0.40 and 0.37 for scorer 1 and 2 respectively). Table VI shows the results of this analysis.

**Table V:** Differences in means between total scores of edited and unedited videos. Student t-test reported for Round 2 Scorer 1 as this was the only round where both final scores were normally distributed.

				95% Confidence Interval of the Difference			
	Difference in Mean*	StDev	Std. Error Mean	Lower	Upper	t	Sig. (2-tailed)
Scorer 1 Round 1	9.9						
Scorer 1 Round 2	4.9	13.3	3.2	-11.8	1.9	-1.5	0.145†
Scorer 2 Round 1	9.9						
Scorer 2 Round 2	7.8						

* Edited minus Unedited, therefore positive values represent a higher mean for Edited videos; †No significant difference between scores (unedited vs edited).

**Table VI:** Correlation between scores of unedited and edited videos for each scorer in the same round.

	Cronbach's alpha*	Intra-class correlation coefficient (ICC)
Scorer 1 Round 1	0.58	0.40
Scorer 1 Round 2	0.88	0.79
Scorer 2 Round 1	0.54	0.37
Scorer 2 Round 2	0.88	0.78
Mean score of both rounds of both scorers	0.92	0.86

All four instances that each video was scored were pooled together and the mean of four total scores calculated for unedited and edited videos separately. There was excellent correlation between the pooled edited and unedited videos (ICC=0.863).

Reviewers 1 and 2 took a total of 139 minutes and 93 minutes respectively to score all unedited videos in their first round. The use of fast-forward at the scorers’ discretion was allowed for scoring unedited videos. Considering the total length of the 17 videos was 9 hours, 53 minutes and 8 seconds, Scorer 1 and 2 watched 23% and 16% of the total length of the unedited footage respectively.

## Discussion

Our study demonstrates high inter-rater and intra- rater reliability for edited videos, but the reviewers were in less agreement about the unedited videos. It is possible that some important parts of the full videos could have been overlooked by one scorer and not the other due to fast-forwarding; something that is less likely when scoring a 4-minute-long edited video without fast-forwarding.

There was also no significant difference between mean scores obtained by edited and unedited videos in any of the rounds for any of the scorers. We would therefore argue that even though it is likely that a self-edited and shortened video will contain the “best” parts of the procedure by deleting mistakes and risky movements, they reflect the surgeon’s overall skills. It is possible that an experienced scorer can judge the surgical skills of an operator by watching only parts of the procedure. Conversely, two studies (Scott et al., 2000a; Datta et al., 2006) have showed poor correlation between edited surgical videos and live assessment or full-length videos.

In our study, edited videos consistently showed better results, both with test-retest reliability and inter-rater reliability. This, and the fact that the inter-rater and intra-rater reliability of GLVS was better for edited videos suggest that edited videos can reliably be used for assessment of surgical skills.

GLVS was found to have a high internal consistency, suggesting that the four components are measuring similar constructs. Some degree of inter-relation is to be expected. For example, bad “tissue handling”, to some extent means a low score in the “safe practice” component too; or if a surgeon scores very high in “movements and coordination” component, it is likely that they also score high in “progress and speed”.

GLVS had a good inter-rater reliability (ICC=0.64) for edited videos, which is comparable to many studies. The videos used in this study included a range of basic gynaecological procedures with varying degrees of complexity, performed with various instruments in different hospitals, whereas most multi-item scoring systems of laparoscopic videos tools are mainly procedure specific. Larsen et al. (2008) used recordings of 21 salpingectomies to validate a procedure-specific rating scale and reported an inter-rater agreement (IRA) of 0.83. These videos were collected prospectively, and all salpingectomies were performed on the right side, using a specific surgical technique using the same instrument. As laparoscopic hysterectomy is the most performed gynaecological procedure, many tools have been developed to assess this procedure. Recently, Crochet et al. (2021) evaluated the validity of a procedure specific rating scale (H-OSAT) designed to assess the operative performances of laparoscopic hysterectomy with or without salpingo-oophorectomy. There was good validity evidence in 3 out of 7 core tasks (creation of bladder flap, colpotomy and closure of vaginal vault) of the H-OSAT rating scale. The author hypothesized that this was due to the 3 tasks requiring a high level of generic skills, good understanding of anatomical landmarks and required a longer operative time when compared to other tasks. Another study (Goderstad et al., 2016) developed a competence assessment tool for laparoscopic supracervical hysterectomy (CAT-LSH) which also showed high inter-rater reliability and had good construct validity when compared to GOALS.

We applied the idea of a multi-item anchored scoring system from the Global Operative Assessment of Laparoscopic Skills (GOALS) scoring system devised by Vassiliou et al. (2005). This system has been shown to have excellent internal consistency (Cronbach’a alpha 0.91-0.93). The five-item score was originally designed by Reznick et al. (1997) for open surgery and was then modified by Vassiliou et al. (2005) for laparoscopic gastrointestinal surgery and validated for a particular part of laparoscopic cholecystectomy.

We changed the 5-point system to an 11-point system partly because the Visual Analogue System is familiar to most gynaecologists due to its use in pain scoring. Furthermore, we anticipate that a wider range may theoretically improve construct validity and may make it a better tool for discriminating between different skill levels. However, we did not aim to measure construct validity in this study because our study subjects were more or less at the same level of experience.

The GOALS system includes anchor points at 1,3 and 5. We used anchor points for 0, 3, 7 and 10 for GLVS. These anchor points can theoretically reduce the likelihood of significant difference between scorers. They define the meaning of each point and are probably more useful in our 11-point system than Vassiliou’s 5-point system.

We felt that the five modules used in the GOALS were likely to have significant overlapping. These were 1. depth perception, 2. bimanual dexterity, 3. efficiency, 4. tissue handling, 5. autonomy. Depth perception and bimanual dexterity are very closely related. Autonomy in the context of scoring videos without watching the surgeon’s behaviour was felt to be vague and difficult to judge.

In developing GLVS, we focused on four primary areas that we believe are central to proficient and safe surgical practice: movement and coordination, progress and speed, safety, and autonomy. The choice of these areas, as well as the respective point allocations, were based on our combined expertise and understanding of surgical practice and education, rather than any pre-existing criteria. Recognizing the importance of patient safety in surgical practice, we assigned “safety” a double weight in the total GLVS score. We chose a multimodular system due its ability to provide both summative and formative assessment and feedback as opposed to an overall score only. This allows the assessor to feedback on specific areas where improvement is required. We believe GLVS offers a balanced and practical approach to surgical skills assessment, even as we acknowledge that no scoring system can be entirely perfect or comprehensive.

In terms of limitations, we recognize that the GLVS, like many other assessment tools, has a subjective component. Phrases such as “little useful progress,” “good hand-eye coordination,” and “difficult to watch” are qualitative assessments rather than strict quantitative measures. Despite this, the use of clear anchor points and multiple raters, as well as the evaluation of inter-rater reliability, aimed to standardize these assessments as much as possible. While future work could focus on refining the descriptors used in GLVS to make them more objective, we acknowledge and accept that a degree of subjectivity, influenced by the assessor’s own experience and expertise, is inevitable and potentially beneficial in the context of surgical skills assessment.

Whilst GLVS offers a practical and accessible tool for assessing surgical skills, we recognize that more objective methods of assessment, such as simulation-based techniques, provide valuable, quantifiable insights into certain aspects of technical skill. These methods, however, are often resource- intensive and may not be widely accessible. Furthermore, they may not fully capture the complex decision-making processes involved in real-world surgical practice. Thus, while GLVS relies more on subjective assessment by experienced surgeons, it complements these more objective methods by providing a holistic assessment of surgical skills that incorporates both technical and decision-making competencies.

In this study, 18 out of the initial 50 videos had to be excluded due to technical issues, resulting in a smaller dataset for our analysis. Future studies with larger sample sizes could further validate our findings. Additionally, while the GLVS tool demonstrated good inter and intra-rater reliability among the assessors in this study, further validation with a wider population of assessors would be beneficial to enhance the generalizability of our findings.

Other methods of assessment for surgical skills using videos have been proposed and tested. Error- based scoring systems use a detailed definition for errors and a list of errors to assist in scoring. Many studies have demonstrated good inter-rater agreement using this method of assessment. Van Sickle et al. (2008) and Husslein et al. (2015) evaluated the validity Generic Error Rating Tool (GERT) on 20 unedited recordings of total laparoscopic hysterectomies. GERT is designed to capture and analyse technical errors. This study showed that GERT had high inter-rater and intra- rater reliability (ICC >0.95) and was the first tool allowing objective error analysis in gynaecologic laparoscopic surgery. These systems, however, are complicated, procedure specific and are perhaps more suitable for bench models, where variables can be minimized.

Although our study did not directly include robotic procedures or more complex surgical operations, we hypothesize that the foundational elements and principles of GLVS could be applicable across all endoscopic procedures, potentially extending to those performed with robotic assistance. This assumption, however, remains theoretical at this stage, and we acknowledge that further empirical validation is needed. It is our belief that GLVS could potentially serve as an assessment tool for gynaecological robotic procedures and more complex operations, but we affirm that extensive validation studies in these contexts are necessary. As such, we caution that our current conclusions should be considered within the scope of our study’s limitations, and we recommend additional research in these areas for more definitive insights.

Finally, while our study focused on assessing the reliability and internal consistency of the GLVS tool, future research could investigate its effectiveness in distinguishing between different levels of surgical expertise. Even though the assessors involved in this study are also authors of the manuscript, they were not part of the development of the GLVS tool. They were chosen specifically for their expertise and were not given any specific training in using the tool prior to the study, reflecting our intention to evaluate the intuitiveness and ease of use of GLVS.

We recognize that the complexity of surgical procedures can vary based on factors such as patient demographics, surgical history, and clinical and medical history. While these variables were not controlled for in this study, it is important to consider them when using GLVS for surgical feedback and learning, as they can influence the perceived competence of the surgeon.

## Conclusion

GLVS is a reliable scoring tool for self-edited shortened videos of laparoscopic gynaecological procedures to assess surgical skills. Edited videos do not seem to score significantly higher or lower compared to their unedited, full-length version and therefore could be used for assessment of surgical skills. GLVS shows considerable promise in aiding surgical skill assessment using objective criteria. It could be used by education bodies or professional societies for assessment and accreditation of surgeons using edited videos of procedures and it allows assessments to be conducted remotely and anonymously.
